# Network-based analysis of calcium-binding protein genes identifies Grp94 as a target in human oral carcinogenesis

**DOI:** 10.1038/sj.bjc.6603948

**Published:** 2007-08-28

**Authors:** H Nomura, K Uzawa, Y Yamano, K Fushimi, T Ishigami, Y Kato, K Saito, D Nakashima, M Higo, Y Kouzu, K Ono, K Ogawara, M Shiiba, H Bukawa, H Yokoe, H Tanzawa

**Affiliations:** 1 Department of Clinical Molecular Biology, Graduate School of Medicine, Chiba University, 1-8-1 Inohana, Chuo-ku, Chiba 260-8670, Japan; 2 Division of Dentistry and Oral-Maxillofacial Surgery, Chiba University Hospital, 1-8-1 Inohana, Chuo-ku, Chiba 260-8670, Japan; 3 Center of Excellence (COE) Program in the 21st Century, Graduate School of Medicine, Chiba University, 1-8-1 Inohana, Chuo-ku, Chiba 260-8670, Japan

**Keywords:** oral squamous cell carcinoma, Ca^2+^-binding protein, pathway analysis, glucose-regulated protein 94 kDa, poor prognosis

## Abstract

To characterise Ca^2+^-binding protein gene expression changes in oral squamous cell carcinomas (OSCCs), we compared the gene expression profiles in OSCC-derived cell lines with normal oral tissues. One hundred Ca^2+^-binding protein genes differentially expressed in OSCCs were identified, and genetic pathways associated with expression changes were generated. Among genes mapped to the network with the highest significance, glucose-regulated protein 94 kDa (Grp94) was evaluated further for mRNA and protein expression in the OSCC cell lines, primary OSCCs, and oral premalignant lesions (OPLs). A significant (*P*<0.001) overexpression of Grp94 protein was observed in all cell lines compared to normal oral epithelium. Immunohistochemical analysis showed highly expressed Grp94 in primary OSCCs and OPLs, whereas most of the corresponding normal tissues had no protein immunoreaction. Real-time quantitative reverse transcriptase-PCR data agreed with the protein expression status. Moreover, overexpression of Grp94 in primary tumours was significantly (*P*<0.001) correlated with poor disease-free survival. The results suggested that Grp94 may have potential clinical application as a novel diagnosis and prognostic biomarker for human OSCCs.

Cytosolic Ca^2+^, a basic parameter of intracellular homeostasis, has a wide variety of important cellular functions, that is, cell growth and proliferation, processing and folding of endoplasmic reticulum (ER)-translated proteins, and Ca^2+^-mediated signalling in response to extracellular stimuli (Berridge *et al*, 2000; Kunzelmann, 2005). Under physiologic conditions, many aspects of these processes are mediated by members of the Ca^2+^-binding protein (Nelson and Chazin, 1998). Since considerable evidence shows that intracellular Ca^2+^ deregulation is crucial for tumour growth and development (Durham and Walton, 1982; Huang *et al*, 2005; Ding *et al*, 2006; Thebault *et al*, 2006), understanding the genetic mechanisms of the Ca^2+^-binding protein genes involved in carcinogenesis is imperative for the development of new therapeutic strategies.

Ca^2+^-binding protein is characterised by the presence of the putative Ca^2+^-binding domain; EF-hand structure is an essential mediator of intracellular Ca^2+^ control elements, such as the plasma membrane Ca^2+^ pumps or ER (Koch *et al*, 1986; Nelson and Chazin, 1998; Haeseleer *et al*, 2000). A number of Ca^2+^-binding proteins are thought to be implicated in establishing the malignant and metastatic phenotypes of various tumours (Chen *et al*, 1997; Takenaga *et al*, 1997; Van Ginkel *et al*, 1998), and recent studies have reported altered expression of several Ca^2+^-binding protein genes in a wide range of human malignancies (Bustin *et al*, 2001; Pietas *et al*, 2002; Lakshmikuttyamma *et al*, 2004; Imazawa *et al*, 2005). Regarding oral squamous cell carcinomas (OSCCs), aberrant expression of S100 Ca^2+^-binding protein families seen in tumour sites has been correlated with cancer cell invasion or metastasis (Moriyama-Kita *et al*, 2004; Tsai *et al*, 2005). We recently identified certain Ca^2+^-binding protein genes differentially expressed in OSCCs compared with normal oral squamous epithelium, including *PMCA1* (Saito *et al*, 2006), *ITPKA* (Kato *et al*, 2006), *ATP2A2* (Endo *et al*, 2004), *CALR* (Shimada *et al*, 2005), and *E-cadherin* (Saito *et al*, 1998).

We recently developed the strategy of using a knowledge-based network approach to search for candidate genes relevant to the treatment of oral cancers (Kasamatsu *et al*, 2005; Higo *et al*, 2006; Ishigami *et al*, 2007). Thus, this study aimed to explore further the global changes in Ca^2+^-binding protein genes associated with OSCCs by applying network development tools to expand on microarray analysis methods. In addition, a candidate gene for therapeutic targeting and follow-up studies was evaluated further for the expression status of the mRNA and protein in a large series of OSCCs and oral premalignant lesions (OPLs).

## MATERIALS AND METHODS

### Tissue specimens

Eighty pairs of primary OSCC samples and corresponding normal oral epithelial tissues or 20 OPLs (diagnosed as oral leukoplakias) were obtained at the time of surgery performed at Chiba University Hospital between 1998 and 2005. All patients provided informed consent according to the protocol that was reviewed and approved by the Institutional Review Board of Chiba University before any procedures were performed. Postoperative follow-up data were collected until April 2006 or until the day of the patient's death, metastasis, or local recurrence. The median follow-up time was 2.1 years (range: 3 months to 8 years).

The resected tissues were divided into two parts: one was frozen immediately after removal of the surrounding normal tissue and stored at −80°C until RNA extraction, and another was fixed in 10% buffered formaldehyde solution for pathologic diagnosis and immunohistochemical staining. Histopathologic diagnosis of each tumour specimen was performed according to the International Histological Classification of Tumours by the Department of Pathology, Chiba University Hospital. Clinicopathologic staging was determined by the TNM classification of the International Union against Cancer. All OSCC samples were histologically confirmed and checked to ensure the presence of tumour in greater than 80% of specimens.

Formalin-fixed, paraffin-embedded human breast cancer tissue (positive control for Grp94 expression) was purchased from Lab Vision Co., Fremont, CA, USA (GRP94, Ab-1, positive control for immunohistochemistry).

### Cell culture

The OSCC-derived cell lines used in this study were HSC-2, HSC-3, Ca9-22 (Human Science Research Resources Bank, Osaka, Japan), H-1, Sa-3 (provided by Dr Fujita, Wakayama Medical University, Wakayama, Japan), and OK-92 (established from carcinoma of the tongue in our department) (Takahashi *et al*, 1989). All OSCC-derived cell lines were cultured in Dulbecco's modified Eagle's medium F-12 HAM (Sigma-Aldrich Co., St Louis, MO, USA) supplied with 10% heat-inactivated fetal bovine serum (Sigma) and 50 U ml^−1^ penicillin and streptomycin (Sigma). The human normal oral keratinocytes (HNOKs) cell line was cultured and maintained in defined keratinocyte-SFM (Gibco BRL, Gaithusberg, Germany). All cell lines were incubated at 37°C in a humidified atmosphere with 5% CO_2_.

### Protein and mRNA extraction

Protein was extracted when the cells reached 80–90% confluence; they were washed two times with phosphate-buffered saline (PBS), scraped into a tube, and centrifuged briefly. The cell pellets were incubated for 30 min in a lysis buffer (LB) containing 7 M urea, 2 M thiourea, 4% w v^−1^ CHAPS, and 10 mM Tris pH 8.0, and lysed by sonication (3 × 10 s pulses on ice). The sample was centrifuged at 13 000 r.p.m. for 20 min. The supernatant containing the cell proteins then was recovered and the protein concentration was measured with a Protein Assay Kit (Bio-Rad Laboratories, Hercules, CA, USA) and adjusted to 1 mg ml^−1^ with LB. The pH of the protein sample was adjusted to 8.5 with 30 mM Tris–HCl. Total RNA was extracted using Trizol reagent (Invitrogen Life Technologies, Carlsbad, CA, USA) according to the manufacturer's instructions. Total protein extracted from a human breast cancer cell line, SKBR3 (positive control for Grp94 expression), was obtained from Lab Vision (GRP94, Ab-1, positive control for Western blotting). Each extracted RNA or protein was stored separately at −80°C until use.

### Affymetrix GeneChip hybridisation

Double-stranded cDNA was synthesised from 20 *μ*g of total RNA using the Superscript Choice system (Invitrogen). After phenol/chloroform extraction and ethanol precipitation, a biotin-labelled *in vitro* transcription reaction was carried out using the cDNA template (Enzo Bioarray, Farmingdale, NY, USA). cRNA (7 *μ*g) was fragmented according to Affymetrix protocols and added to the recommended hybridisation mixture. Expression profiles were created using the Human Genome U 133 Plus 2.0 arrays containing 54 675 probe sets (Affymetrix, Santa Clara, CA, USA). Arrays were stained with phycoerythrin–streptavidin, and the signal intensity was amplified by treatment with a biotin-conjugated anti-streptavidin antibody followed by a second staining with phycoerythrin–streptavidin. Arrays stained a second time were scanned using the Affymetrix GeneChip Scanner 3000 (Affymetrix). Expression data were analysed using GeneChip Operating Software 1.1 (Affymetrix), and Ca^2+^-binding protein genes were then classified by GeneSpring 6.1 (Silicon Genetics, Redwood City, CA, USA).

### Network and gene ontology analysis

A list of Ca^2+^-binding protein genes identified by microarray analysis was used for network and gene ontology analyses. Gene accession numbers were imported into the Ingenuity Pathway Analysis (IPA) Software (Ingenuity Systems, Mountain View, CA, USA). The IPA database consists of proprietary ontology representing 300 000 biologic objects ranging from genes, proteins, and molecular and cellular processes. More than 11 200 human genes are currently represented in the database. The genes were categorised based on location, cellular components, and reported or suggested biochemical, biologic, and molecular functions using the software. The identified genes also were mapped to genetic networks available in the Ingenuity database and then ranked by score. The score is the probability that a collection of genes equal to or greater than the number in a network could be achieved by chance alone. A score of 3 indicates a 1/1000 chance that the focus genes are in a network due to random chance. Therefore, a score of 3 or higher has a 99.9% confidence level of not being generated by random chance alone. This score was used as the cutoff for identifying gene networks.

### Western blot analysis

Among genes identified, glucose-regulated protein 94 kDa (Grp94) was selected for further analyses. We carried out Western blot analysis to investigate Grp94 protein expression in normal oral epithelium and OSCC-derived cell lines. Protein extracts were electrophoresed on 11% sodium dodecyl sulphate-polyacrylamide gel electrophoresis gels, transferred to polyvinylidene difluoride (PVDF) membranes (Bio-Rad Laboratories, Hercules, CA, USA), and blocked for 1 h at room temperature in 5% skim milk. Immunoblot PVDF membranes were washed with 0.1% Tween-20 in TBS (TBS-T) five times, and 2 *μ*g ml^−1^ affinity-purified rat antihuman Grp94 monoclonal antibody (Lab Vision) was added directly to the TBS-T solution for 2 h at room temperature. The PVDF membranes were washed again and incubated with 1 : 10 000 ratio of horseradish peroxidase (HRP)-conjugated antirat IgG Ab (Santa Cruz Biotechnology, Santa Cruz, CA, USA) as a secondary antibody for 20 min at room temperature. The membranes were then incubated with enhanced chemiluminescence (ECL) +HRP substrate solution included in the ECL+ kit (Amersham Biosciences, Buckinghamshire, UK), and immunoblotting was visualised by exposing the membrane to Hyperfilm (Amersham Biosciences). Triplicate examinations were performed to confirm the specificity of the antibody.

### Immunofluorescence

The cells grown on glass coverslips were washed with PBS, fixed in 4% paraformaldehyde for 10 min at 37°C followed by absolute methanol for 10 min at 4°C, and blocked in PBS containing 1% skimmed milk for 10 min. The samples then were incubated with anti-Grp94 antibody (Lab Vision) at a dilution of 1 : 100 for 2 h, rinsed two times with PBS, and incubated with goat antirat secondary antibody labelled with Alexa Fluor 546 (Molecular Probes, Leiden, The Netherlands) for 1 h. For counterstaining of the nucleus, the dishes were then incubated with 1 *μ*g ml^−1^ Cellstain-DAPI (Dojindo Laboratories, Kumamoto, Japan) in PBS for 10 min. The samples were observed under a Leica TCS2-MP confocal system (Leica Laserteknik, Mannheim, Germany) and Coherent Mira tunable pulsed titanium sapphire laser (Coherent Laser Group, Santa Clara, CA, USA).

### Immunohistochemistry

To examine the cellular distribution of Grp94 protein in normal and OSCCs, we carried out immunohistochemical staining on 4-*μ*m sections of paraffin-embedded specimens. Briefly, after deparaffinisation and hydration, the slides were pretreated in 10 mM sodium citrate buffer (pH 6.0) in a microwave oven for 5 min at 95°C. The endogenous peroxidase activity was quenched by 30-min incubation in a mixture of 0.3% hydrogen peroxide solution in 100% methanol. After being washed with PBS buffer, sections then were incubated with primary antibody antiGrp94 antibody (1 : 100 dilution) at room temperature in a moist chamber for 2 h. After being washed with PBS buffer, the slides were treated with biotinylated secondary antibody for 1 h followed by colour development in 3,3′-diaminobenzidine tetrahydrochloride (Dako Japan Inc., Kyoto, Japan). Finally, the slides were lightly counterstained with haematoxylin. A known positive breast cancer control section for Grp94 was clearly stained. While the breast cancer cells were immunohistochemically positive for Grp94, those of the negative control prepared by omitting the primary antibody were negative, thus confirming the staining specificity. To quantitate the state of the Grp94 protein expression, the mean percentage of positive tumour cells was determined in at least five random fields at × 400 magnification in each section. The intensity of the Grp94 immunoreaction was scored as follows: 1+, weak; 2+, moderate; and 3+, intense. The percentage of positive tumour cells and the staining intensity were then multiplied to produce a Grp94-immunohistochemical staining score. Cases with a Grp94-immunohistochemical staining score exceeding 65.56 (maximum score of normal tissues) were considered positive. Two independent pathologists, neither of whom had knowledge of the patients' clinical status, made these judgments.

### mRNA expression analysis

The expression levels of *Grp94* mRNA were examined in 50 OSCC specimens from patients with primary tumours among the OSCC cases studied by immunohistochemical staining. Control reactions were prepared in parallel without reverse transcriptase (RT). Before cDNA synthesis, residual genomic DNA was removed from the total RNA using DNase I treatment (DNA-free; Ambion, Austin, TX, USA). The primer sequences used to analyse *Grp94* mRNA expression were 5′-AGCAAGACGTGTTCGATTC-3′ (nucleotides 1392–1410) and 5′-CCTCAATTTTGTCAAGGGTG-3′ (nucleotides 1607–1626). The sequences of specific primers were checked before use to avoid amplification of genomic DNA or pseudogenes by the Primer3 program (available at http:www-genome.wi.mit.edu/cgi-bin/primer/primer3_www.cgi). Amplified products were analysed by 3% agarose gel electrophoresis to ascertain size and purity. Real-time quantitative RT (qRT)–PCR was performed with a single method using the LightCycler FastStart DNA Master SYBR Green I kit (Roche, Mannheim, Germany). To prepare the standard curve, 3 *μ*g of total RNA from normal oral tissue was reverse-transcribed with Superscript RT (Life Technologies, Grand Island, NY, USA) and oligo-d(T)12-18 primer, after which serial dilutions were made corresponding to cDNA transcribed from 300, 30, 3.0, and 0.3 ng of total RNA. PCRs using LightCycler apparatus were carried out in a final volume of 20 *μ*l of reaction mixture consisting of 2 *μ*l of FirstStart DNA Master SYBR Green I mix, 3 mM MgCl_2_, and 0.2 *μ*l of primers, according to the manufacturer's instructions. The reaction mixture then was loaded into glass capillary tubes and submitted to an initial denaturation at 95°C for 10 min, followed by 45 rounds of amplification at 95°C 10 s) for denaturation, 58°C (10 s) for annealing and 72°C for extension, with a temperature slope of 20°C s^−1^, performed in the LightCycler. The transcript amount for *Grp94* was estimated from the respective standard curves and normalised to the *GAPDH* transcript amount determined in corresponding samples.

### Statistical analysis

Differences in gene expression levels between Grp94-positive and Grp94-negative cases were calculated with the Mann–Whitney's *U*-test. Correlations between Grp94-immunohistochemical staining scores and clinicopathologic features were evaluated by Fisher's exact test. Overall survival time was defined as the interval between the date of treatment and the date of death or until the last objective follow-up information was obtained. Disease-free survival time was regarded as the time interval between tumour treatment and detection of the first locoregional recurrence, distant metastasis, or both or the date of the last follow-up, whichever occurred first. Patients without evidence of disease (local recurrence or metastasis) during follow-up were considered to have a good prognosis; patients with local recurrence, distant metastasis during follow-up were regarded as having a poor prognosis. Survival curves were obtained by the Kaplan–Meier method and differences in survival rates between Grp94-positive and Grp94-negative cases were compared by log-rank tests with 95% significance. The criterion for statistical significance was *P*<0.05. The data are expressed as the mean values±s.e.

## RESULTS

### Altered Ca^2+-^binding protein genes expression in OSCC-derived cell lines

To investigate the expression profile changes of Ca^2+^-binding protein genes in OSCC, we initially performed oligonucleotide microarray analyses using the Affymetrix U133A chips, which contained 54 675 probe sets with RNAs isolated from four OSCC-derived cell lines (HSC-2, HSC-3, H-1, and Sa-3). Control RNAs consisted of a pool made of normal tongue tissue RNAs extracted from three patients. Expression data were analysed using the GeneChip Operating Software 1.1 (Affymetrix) and GeneSpring 6.1 (Silicon Genetics). Of the 2241 Ca^2+^-binding protein gene transcripts analysed, 100 (4.4%) were identified as genes that were differentially expressed at least two-fold in all OSCC-derived cell lines examined compared with controls. Of these, 24 genes were found to be upregulated and 76 genes were downregulated in OSCC (data not shown).

### Network and gene ontology analysis

**Figure 1:**
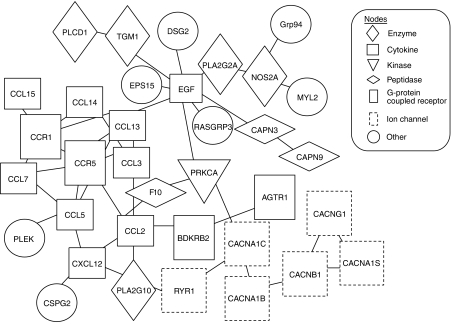
Network with the highest score (network 1). Expression levels of all 35 genes (100%) are altered significantly in the oral squamous cell carcinomas (OSCCs). Functional relationships between gene products based on known interactions in ingenuity pathway analysis (IPA) knowledge are described.

On the basis of all genes identified as described previously (referred to as focus genes), new and expanded pathway maps and connections and specific gene–gene interactions were inferred, functionally analysed, and used to build on the existing pathway using the IPA knowledge base. To generate networks, the knowledge base was queried for interactions between focus genes and all other gene objects stored in the database. Six networks were found to be significant in OSCC in that they had more of the identified genes present than would be expected by chance (Table 1). Of them, the network with the highest score (network 1, score=71) was generated with all focus genes (Figure 1; Table 2). We also performed gene ontology analyses of 100 focus genes using the IPA tool. Seventy-six upregulated genes were associated with cancer-related functions, cell death, and the cell cycle; 24 downregulated genes were associated with cellular movement, signalling, and function and maintenance (Table 3). In particular, we found that the cellular signalling was contained in both the upregulated and downregulated groups. Six upregulated genes were associated with a variety of cellular signalling functions, including induction/production of nitric oxide, inhibition of cyclic adenosine monophosphate, and inactivation of mitogen-activated protein kinase. In contrast, 31 downregulated groups played a critical role in cellular signalling, which is related to intracellular Ca^2+^ homeostasis, suggesting that genes associated with cell signalling may act in a different manner during development of OSCC.

Among genes mapped to the network with the highest significance, *Grp94* was analysed further.

### GRP94 expression in OSCCs

**Figure 2:**
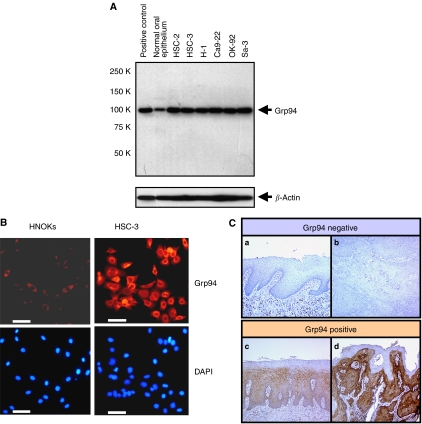
Representative results of expression of Grp94 protein in oral squamous cell carcinomas (OSCC)-derived cell lines. (**A**) Western blot analysis of Grp94 protein in OSCC-derived cell lines and normal oral epithelium. All OSCC-derived cell line extracts exhibit a single band for Grp94 protein expression at high levels. In contrast, normal oral epithelium shows a low level of Grp94 protein expression. (**B**) Immunocytochemical analysis shows strong immunoreactivity of Grp94 in an OSCC-derived cell line (HSC-3) compared with human normal oral keratinocytes (HNOKs). DAPI staining was used to stain DNA. Bar, 100 *μ*m. (**C**) Immunohistochemical staining of Grp94 in normal tissue, oral premalignant lesion (OPL), and primary OSCC. (**a**) Normal oral tissue exhibits negative Grp94 protein expression. (**b**) Grp94-negative case of OSCC. (**c**) Grp94-positive case of OPL. The immunoreaction is enhanced in the spinous layer. (**d**) Grp94-positive case of OSCC. Strong positive immunoreactivity for Grp94 is detected in the cytoplasm.

The state of Grp94 protein expression in six OSCC-derived cell lines (HSC-2, HSC-3, H-1, Sa-3, Ca9-22, and OK-92) (*n*=6) was evaluated by Western blot analysis. Figure 2A shows representative results. The band size was found to be 94 kDa as a single band, as reported by Little *et al* (1994). A significant (*P*<0.001) overexpression of Grp94 protein was observed in all OSCC-derived cell lines compared with normal oral epithelium. We also assessed the level of Grp94 protein expression in an OSCC-derived cell line (HSC-3) and HNOKs by immunofluorescence analysis. Representative cases of immunofluorescence are shown in Figure 2B. Strong immunoreactivity of Grp94 protein was detected in the cytoplasm of the OSCC-derived cell line HSC-3 compared with the HNOKs.

Immunohistochemical staining was performed using a series of surgical OSCC specimens, including 80 OSCCs with corresponding normal tissues and 20 OPLs. Grp94 overexpression was found not only in OSCCs (*P*<0.001) but also in OPLs (*P*<0.001). Among the OSCCs, 46 had significantly increased expression of Grp94 (immunohistochemical staining score, >65.56). In contrast, the normal tissues had no or significant downregulation of Grp94 expression and were considered Grp94 negative (Fisher's exact test). There was no significant difference between the frequency of Grp94-positive cases and clinicopathologic features (Table 4).

**Figure 3:**
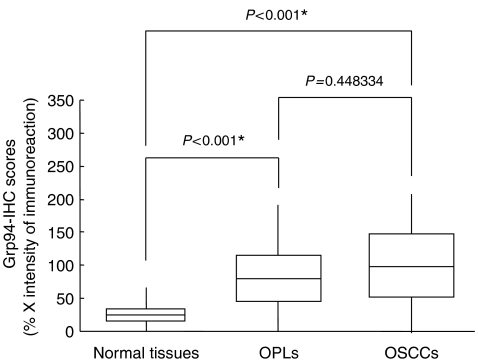
State of Grp94 protein expression in normal tissues (*n*=80), oral premalignant lesions (OPLs) (*n*=20) and primary oral squamous cell carcinomas (OSCCs) (*n*=80). Grp94 protein expression in OPLs and OSCCs is significantly higher than in normal oral tissues (*P*<0.001, Mann–Whitney's *U*-test). The results are expressed as the mean±s.d.

Thirteen of 20 (65%) OPLs were defined as Grp94 positive. The Grp94-immunohistochemical staining scores for normal tissues, OPLs, and OSCCs, respectively, ranged from 0 to 65 (mean, 12.173), 12 to 191 (mean, 85.27), and 0 to 262 (mean, 106.511) (Figure 2C). Grp94 expression levels in primary OSCCs and OPLs were significantly (*P*<0.001) higher than those in normal oral tissues (Figure 3). In contrast, we found no significant (*P*=0.448334) difference in Grp94-immunohistochemical staining scores between OSCCs and OPLs.

**Figure 4:**
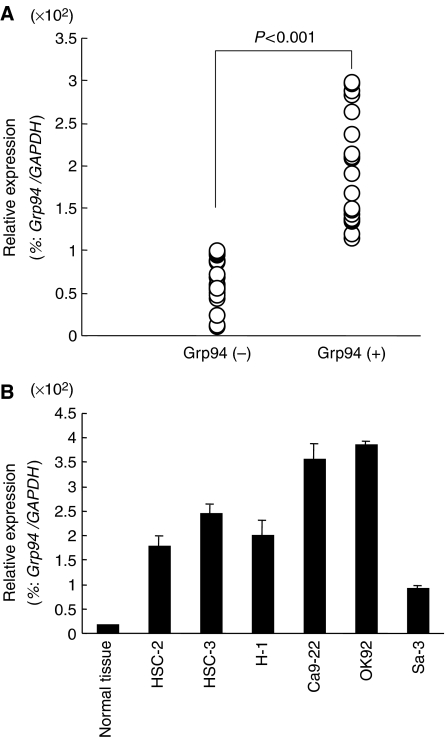
*Grp94* mRNA expression status in primary oral squamous cell carcinomas (OSCCs) and OSCC-derived cell lines. (**A**) Comparison of *Grp94* mRNA expression levels between Grp94-positive and Grp94-negative cases classified by immunohistochemical analysis. There is a significant difference in the *Grp94* mRNA expression levels between the negative and positive cases (*P*<0.001, Mann–Whitney's *U*-test). (**B**) Quantification of mRNA levels in OSCC-derived cell lines by qRT–PCR analysis. Significant upregulation of *Grp94* mRNA expression is seen in all OSCC-derived cell lines examined compared to normal oral epithelium. Data are expressed as the means±s.d.

Grp94 mRNA expression levels were significantly upregulated in primary tumours of randomly selected Grp94-positive cases (*n*=29) compared to selected Grp94-negative cases (*n*=21, Mann–Whitney *U-*test, *P*<0.001; Figure 4A). mRNA expression levels were normalised to *GAPDH*. The relative mRNA expression levels in positive and negative cases ranged from 113 to 297 (mean, 191.3) and 9 to 88 (mean, 56.6), respectively. Grp94 expression was upregulated significantly in all OSCC cell lines examined compared to normal oral epithelium used as a control (Figure 4B).

### Prognostic significance of GRP94 expression in OSCCs

To assess whether Grp94 expression also had a prognostic impact on patients with OSCC, clinical postoperative data from patients whose tissue samples were investigated for Grp94 protein expression by immunohistochemical analysis were statistically analysed. Tumours with significantly increased expression levels of Grp94 protein (immunohistochemical staining score, >65.56; maximum score of normal tissues) were defined as Grp94 positive (*n*=46). Cases with no or significant downregulation of Grp94 protein expression (immunohistochemical staining score, <65.56) were considered Grp94 negative (*n*=34).

**Figure 5:**
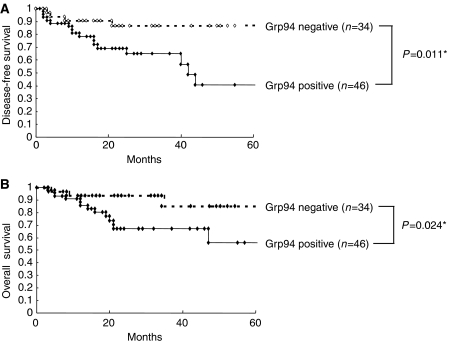
Kaplan–Meier curves for disease-free (**A**) or overall survival (**B**) according to Grp94-positive expression in patients with oral squamous cell carcinoma (OSCC) (log-rank test (**A**), *P*=0.011 (**B**), *P*=0.024). Cases with significantly increased expression of Grp94 protein (immunohistochemical staining score, >65.56; maximum score of normal tissues) are considered Grp94 positive. Overexpression of Grp94 protein is significantly associated with a poor outcome in patients with OSCC.

Survival curves in relation to Grp94 protein expression are shown in Figure 5. Log-rank survival analysis indicated that Grp94-positive expression was significant both for disease-free survival (*P*=0.011; Figure 5A) and overall survival (*P*=0.024; Figure 5B), which suggested that high expression of Grp94 protein in patients with OSCCs was significantly associated with a poor prognosis.

## DISCUSSION

Using a microarray technique, we identified a total of 100 Ca^2+^-binding protein genes aberrantly expressed in OSCC cells. Pathway analysis could further characterise six networks from the 100 genes (Table 1). In addition, the network of the highest significance was generated entirely from 35 of the 100 focus genes (Figure 1), including a number of the *CCL* and *CXC* families. Previous studies have shown that chemokine-mediated JAK/STAT activation is critical for phospholipase C-*β* dependent Ca^2+^ flux (Li *et al*, 2000; Lewis, 2001; Thelen, 2001; Soriano *et al*, 2003). This evidence together with the current results suggests that reduced expression of the main chemokine families, *CCL* and *CXC*, disrupts intracellular Ca^2+^ homeostasis and initiates oral tumorigenesis. The network with the highest significance also contained several cancer-related genes with high expression levels, including *DSG2*, *PRKCA*, *PLA2G10*, and *Grp94*.

Considerable evidence has shown a significant association between the heat-shock protein 90 (Hsp90) and a wide range of human malignancies, including head and neck cancers (Ito *et al*, 1998; Baker *et al*, 2005; Yin *et al*, 2005). Grp94, also known as gp96, is the ER-resident member of the Hsp90 family constitutively expressed in virtually all cell types (Van *et al*, 1989) and the most abundant ER chaperon protein showing high homology (50%) to cytosolic counterpart Hsp90 (Sorger and Pelham, 1987). Increased expression of Grp94 both at the mRNA and protein levels also has been reported in several types of human cancers, such as oesophageal cancer (Wang *et al*, 2005b), lung cancer (Wang *et al*, 2005a), breast cancer (Gazit *et al*, 1999), liver cancer (Lim *et al*, 2005), and colon cancer (Wang *et al*, 2005c). We hypothesised that Grp94 also has potential as an emerging therapeutic target of interest for the treatment of oral cancer. However, the status of Grp94 in OSCC remains unclear and, therefore, we selected it for further investigation.

To confirm our hypothesis, we determined the protein/mRNA expression in a series of OSCC-derived cell lines and human primary OSCCs using immunofluorescence, Western blot analysis, qRT–PCR, and immunohistochemistry. Significant increases in Grp94-protein- and mRNA-expression levels were observed in the OSCC-derived cell lines examined compared with the normal oral epithelium. We also detected a comparatively strong tumour cell-localised cytoplasmic Grp94-immunoreaction in primary OSCCs. By evaluating the Grp94 immunohistochemistry scores, significant upregulation was evident in the primary OSCCs compared with normal tissues. While we could not find a significant correlation between Grp94 protein expression status and any clinicopathologic features examined, high levels of Grp94 protein expression was detected even in the OPLs examined. In addition, the current study showed that Grp94 overexpression is closely related to the disease-free/overall survival (*P*=0.011 and 0.024, respectively). Thus, we suggest that Grp94 could be associated with not only early-stage OSCC development but also tumour prognosis.

Interestingly, a recent study has shown that inhibition of Grp94 expression by geldanamycin in chronic lymphocytic leukaemia cells induces apoptosis with modest cytoprotective effects of primary haematopoietic progenitors from normal bone marrow (Jones *et al*, 2004). In addition, targeting cancer cells with an antisense or RNAi procedure against Grp94 has shown increased chemosensitivity or radiosensitivity (Reddy *et al*, 1999; Kubota *et al*, 2005). From the therapeutic standpoint, this evidence may provide a novel/effective approach for treating human OSCCs.

In summary, we found novel specific networks of Ca^2+^-binding protein genes in OSCC cells and identified several candidate genes for molecular targeting, especially for *Grp94*. Our findings may contribute to an understanding of key biologic functions and pathways of certain Ca^2+^-binding protein genes associated with OSCC and should stimulate further investigation into Ca^2+^-binding protein genes relevant to oral carcinogenesis.

## Figures and Tables

**Table 1:** Genetic networks in the OSCC-derived cell lines

**Network**	**Genes in ingenuity networks^a^**	**Function**	**Score^b^**
1	***ATGR1***, ***BDKRB2***, ***CACNA1B***, ***CACNA1C***, ***CACNA1S***, ***CACNB1***, ***CACNG1***, ***CAPN3***, ***CAPN9***, ***CCL2***, ***CCL3***, ***CCL5***, ***CCL7***, ***CCL13***, ***CCL14***, ***CCL15***, ***CCR1***, ***CCR5***, ***CSPG2***, ***CXCL12***, ***DSG2***, ***EGF***, ***EPS15***, ***F10***, ***MYL2***, ***NOS2A***, ***PLA2G10***, ***PLA2G2A***, ***PLCD1***, ***PLEK***, ***PRKCA***, ***RASGRP3***, ***RYR1***, ***TGM1***, ***Grp94***	Cell signalling, cellular function, and maintenance	71
2	*AGT1*, ***ANXA1***, *ATF1*, ***CALML3***, ***CDH11***, *COPS5*, *DIABLO*, ***DGS3***, *FGF10*, *FPR1*, *IL11*, *IR1RN*, *INHBA*, ***LTBP2***, ***MGP***, ***MMP8***, *MMP13*, *MTPN*, ***NOTCH4***, ***PCDH7***, *RXRA*, ***S100A7***, ***S100A8***, ***S100A9***, *SAA1*, *SCARB1*, *SCUBE1*, ***SCUBE9***, *SERPINA3*, *SOD1*, *TNF*, *TNFRSF4*, *TNFRSF9*, ***TPO***, ***UMOD***	Lipid metabolism, molecular transport	20
3	*AKT1*, ***ATP2A3***, *BAG3*, *BBC3*, *BCL2*, ***CALU***, ***CCL19***, ***CCR7***, ***CDH5***, *CHGB*, ***CHP***, ***CIB1***, *CKS2*, *CLCN3*, *E2F4*, ***EGF***, *ETS1*, ***ITPR3***, *JUB*, *KRAS*, ***LOCG3928***, *MIG-6*, ***MYL1***, ***PPP2R3A***, ***PRKCSH***, *RALGDS*, ***RASGRP1***, *RBL1*, *ROCK1*, *RPS6KB2*, *RRM1*, *RRM2*, ***SLC3A2***, *SLC9A1*, *TERT*	Cancer, cell morphology, cellular compromise	20
4	*AHNAK*, *A****TP2A1***, *BBC3*, *BGN*, ***BMP1***, ***CANX***, ***CCR6***, *CDKN1B*, ***CHGA***, *CHRD*, *COL5A1*, *CTSD*, ***CUBN***, *DDX5*, *DMP1*, ***FBLN5***, ***FREQ***, *GFAP*, *GHR*, *IER3*, *IGF1*, *INPPL1*, *LGALS3*, *LOX*, *LPL*, *METAP2*, ***MRC1***, *MTPN*, *MYC*, ***PLS1***, ***S100A1***, ***S100B***, *SLC2A4*, ***TLL1***	DNA replication, recombination, and developmental disorder, carbohydrate	17
5	*C2*, *C3*, *C1QA*, ***C1S***, ***C3AR1***, ***CCL2***, ***DSC1***, ***DSG1***, ***F13A1***, *HOXD3*, *IGFBP2*, *IGFBP5*, *IL13*, ***ITIH1***, *JUP*, *KLK5*, *MMP2*, *MMP13*, *MMP14*, *MYOD1*, *PROC*, *RP6SKA2*, *S100A4*, *SERPINA1*, ***SLIT2***, ***SPOK3***, *TGM2*, *THBS1*, ***THBS2***, *TIMP1*, ***TKT***, ***TNNC1***, *TNNC2*, *TNNI1*, *TNNT2*	Organism, injury and abnormalities, cardiovascular system	17
6	***ACTN2***, ***ACTN3***, *CALM1*, ***CASQ1***, *CD47*, *CDK6*, *CDKN1A*, *CTSK*, ***CXCL12***, *DCN*, *ERBB2*, *ERBB3*, *ERBB4*, *EREG*, ***FBLN2***, *FBN1*, *GHR*, *GRB7*, *GRK1*, *HAS2*, *HBEGF*, ***HRC***, *IFNA2*, *IL6R*, *MTPN*, ***MYL9***, *MYOZ1*, *PTGS1*, ***RCV1***, *S100A4*, *SERPINA3*, ***TGM1***, *TIMP3*, *TNC*, *TRDN*	Cellular growth and proliferation, cellular movement, cell death	17

a Genes in boldface were identified by microarray analysis to be expressed differentially more than two-fold in OSCCs. Other genes were either not on the expression array or not significantly regulated.

b A score>3 was considered significant.

**Table 2:** Thirty-five focus genes in network 1

**Affymetrix no.**	**Gene**	**Molecular function**	**Location**	**Fold change^a^**
217901_at	DSG2	Cell adhesion	Plasma membrane	85.187
200599_s_at	Grp94	Protein folding and sorting, antigen presentation	Plasma membrane	5.479
210037_s_at	NOS2A	Nitric oxide synthase activity	Cytoplasm	5.072
1560074_at	PRKCA	Protein kinase C activity	Cytoplasm	3.781
210641_at	CAPN9	Calpain activity	Unknown	3.003
207162_s_at	CACNA1B	Voltage-gated calcium channel activity	Plasma membrane	2.894
207222_at	PLA2G10	Unknown	Extracellular space	2.749
207225_at	EPS15	Protein binding	Plasma membrane	0.4539
201511_at	BDKRB2	Receptor activity	Plasma membrane	0.4464
205434_s_at	CACNA1C	Voltage-gated calcium channel activity	Plasma membrane	0.4288
218075_at	AGTR1	Receptor activity	Plasma membrane	0.3305
1558450_at	CCR1	Chemokine receptor activity	Plasma membrane	0.2580
207798_s_at	CACNB1	Voltage-gated calcium channel activity	Plasma membrane	0.2346
228767_at	CCL13	Chemokine receptor activity	Extracellular space	0.2200
235070_at	PLEK	Reorganisation	Cytoplasm	0.1697
229819_at	CCR5	Chemokine receptor activity	Plasma membrane	0.1640
228376_at	CCL7	Chemokine receptor activity	Extracellular space	0.1611
218529_at	F10	Peptidase activity	Extracellular space	0.1583
224938_at	PLA2G2A	Protein binding	Extracellular space	0.1339
224939_at	CACNA1S	Voltage-gated calcium channel activity	Plasma membrane	0.1256
214711_at	CCL5	Chemokine receptor activity	Extracellular space	0.1172
214319_at	CCL3	Chemokine receptor activity	Extracellular space	0.1071
1555904_at	CCL15	Chemokine receptor activity	Extracellular space	0.0868
209665_at	RASGRP3	Protein binding	Cytoplasm	0.0810
220781_at	CACNG1	Voltage-gated calcium channel activity	Plasma membrane	0.0744
1553086_at	CSPG2	Unknown	Extracellular space	0.0736
212698_s_at	EGF	Protein binding	Extracellular space	0.0611
226627_at	PLCD1	Unknown	Cytoplasm	0.0381
213666_at	CXCL12	Chemokine receptor activity	Extracellular space	0.0350
211890_x_at	CAPN3	Calpain activity	Cytoplasm	0.0227
216598_s_at	CCL2	Chemokine receptor activity	Extracellular space	0.0198
235340_at	CCL14	Chemokine receptor activity	Extracellular space	0.0165
205485_at	RYR1	Voltage-gated calcium channel activity	Plasma membrane	0.0126
206008_at	TGM1	Cell formation	Plasma membrane	0.0109
209742_s_at	MYL2	Motor activity	Cytoplasm	0.0100

AGTR1=angiotensin II receptor, type 1; BDKRB2=bradykinin receptor B2; CACNA1B=calcium channel, voltage-dependent, L type, *α* 1B- subunit; CACNA1C=calcium channel, voltage-dependent, L type, *α* 1C subunit; CACNA1S=calcium channel, voltage-dependent, L type, *α* 1S subunit; CACNB1=calcium channel, voltage-dependent, *β* 1 subunit; CACNG1=calcium channel, voltage-dependent, *γ* subunit 1; CAPN3=calpain 3, (p94); CAPN9=calpain 9; CCL2=chemokine (C-C motif) ligand 2; CCL3=chemokine (C-C motif) ligand 3; CCL5=chemokine (C-C motif) ligand 5; CCL7=chemokine (C-C motif) ligand 7; CCL13=chemokine (C-C motif) ligand 13; CCL14=chemokine (C-C motif) ligand 14; CCL15=chemokine (C-C motif) ligand 15; CCR1chemokine (C-C motif); CCR5=chemokine (C-C motif) receptor 5; CSPG2=chondroitin sulfate proteoglycan 2; CXCL12=chemokine (C-X-C motif) ligand 12; DSG2=desmoglein 2; EGF=epidermal growth factor; EPS15=epidermal growth factor receptor pathway substrate 15; F10=coagulation factor X; Grp94=glucose regulated protein, 94 kDa; MYL2=myosin, light polypeptide 2, regulatory, cardiac, slow; NOS2A=nitric oxide synthase 2A; PLA2G2A=phospholipase A2, group IIA; PLA2G10=phospholipase A2, group X; PLCD1=phospholipase C, delta 1; PLEK=pleckstrin; PRKCA=protein kinase C, *α*; RASGRP3=RAS guanyl releasing protein 3; RYR1=ryanodine receptor 1; TGM1=transglutaminase 1.

a Fold overexpression for microarray data of OSCC-derived cell lines compared to normal control.

**Table 3:** Functional characterisation of Ca^2+^-binding protein genes altered expressed in OSCC-derived cell lines

**Relevant function and disease**	***P*-value**	**Gene, no^a^**
*Top-10 functions associated with upregulated genes in OSCC-derived cell lines*		
Cancer^b^	1.15E−3 to 4.27E−2	7
Cell death	1.15E−3 to 6.42E−2	4
Cell cycle	1.86E−3 to 6.95E−2	6
Cell-to-cell signalling and interaction	3.94E−3 to 6.16E−2	7
Cell signalling	3.29E−3 to 6.42E−2	6
Molecular transport	4.29E−3 to 7.47E−2	5
Small molecule biochemistry	4.29E−3 to 7.47E−2	9
Nucleic acid metabolism	5.07E−3 to 2.89E−2	4
Cellular growth and proliferation	5.07E−3 to 6.95E−2	3
Cell morphology	8.24E−3 to 6.95E−2	1
		
*Top-10 functions associated with downregulated genes in OSCC-derived cell lines*		
Cellular movement^c^	1.60E−13 to 8.53E−3	33
Cellular signalling	1.52E−11 to 8.53E−3	31
Cellular function and maintenance	1.52E−11 to 8.53E−3	16
Small molecule biochemistry	1.52E−11 to 8.53E−3	20
Vitamin and mineral metabolism	1.52E−11 to 7.02E−3	32
Tissue morphology	2.10E−11 to 8.53E−3	33
Molecular transport	2.69E−11 to 8.53E−3	18
Skeletal and vascular system development and function	2.08E−10 to 8.53E−3	21
Cancer	5.13E−9 to 8.53E−3	27
Cell-to-cell signalling and interaction	1.01E−8 to 8.53E−3	26

a Number of associated genes.

b Apoptosis, cell movement, proliferation, cell cycle progression, migration, binding quantity of cancer cell line.

c Chemotaxis, migration, cell movement, mobilisation of haematologic cyte.

**Table 4:** Correlation between Grp94 expression and clinical classification in OSCCs

		**Result of immunostaining: number of patients(%)**		
**Clinical classification**	**Total**	**Grp94 (−)**	**Grp94 (+)**	***P*-value^a^**
*Age at surgery (years)*				
<60	23	8 (30)	15 (70)	
60⩽, <70	26	11 (42)	15 (58)	0.431974
70⩽	31	15 (48)	16 (52)	
				
*Gender*				
Male	33	13 (39)	20 (61)	0.654387
Female	47	21 (45)	26 (55)	
				
*T-primary tumour*				
T1	5	2 (40)	3 (60)	
T2	35	15 (43)	20 (57)	0.55542
T3	17	5 (29)	12 (71)	
T4	23	12 (52)	11 (48)	
				
*N-regional lymph node*				
N(−)	52	23 (44)	29 (56)	0.813185
N(+)	28	11 (39)	17 (61)	
				
*Stage*				
I	5	2 (40)	3 (60)	
II	16	7 (44)	9 (56)	0.756529
III	17	7 (41)	10 (59)	
IV	52	28 (54)	24 (46)	
				
*Histopathologic type*				
Well differentiated	51	24 (47)	27 (53)	
Moderately differentiated	22	9 (41)	13 (59)	0.300615
Poorly differentiated	7	1 (14)	6 (86)	
				
*Tumour site*				
Tongue	34	12 (35)	22 (65)	
Gingiva	29	13 (45)	16 (55)	0.90283
Oral floor	7	3 (43)	4 (57)	
Buccal mucosa	6	3 (50)	3 (50)	
Oropharynx	3	2 (67)	1 (33)	
Lip	1	0 (0)	1 (100)	
Leukoplakia	20	7 (35)	13 (65)	—

a *P*<0.05 was considered significant.
